# Musculoskeletal Injuries and Training Patterns in Junior Elite Orienteering Athletes

**DOI:** 10.1155/2015/259531

**Published:** 2015-07-15

**Authors:** Lilian Roos, Wolfgang Taube, Peter Zuest, German Clénin, Thomas Wyss

**Affiliations:** ^1^Swiss Federal Institute of Sport Magglingen (SFISM), 2532 Magglingen, Switzerland; ^2^Department of Medicine, Movement and Sport Science, University of Fribourg, 1700 Fribourg, Switzerland; ^3^Swiss Orienteering Federation, 4600 Olten, Switzerland

## Abstract

Findings about the relation between musculoskeletal injuries and training patterns in orienteering athletes are sparse. Therefore, the musculoskeletal injuries and training patterns of 31 Swiss elite orienteering athletes aged 18-19 years were analyzed in a retrospective study. Individual training diaries and medical records were used to assess training data and injury history, respectively. Group comparisons and a multiple linear regression (MLR) were performed for statistical analysis. The junior elite orienteering athletes performed 7.38 ± 2.00 training sessions weekly, with a total duration of 455.75 ± 98.22 minutes. An injury incidence rate (IIR) of 2.18 ± 2.13 injuries per 1000 hours of training was observed. The lower extremity was affected in 93% of all injuries, and the knee (33%) was the most commonly injured location. The MLR revealed that gender and six training variables explained 60% of the variance in the injury severity index in this study. Supported by the low IIR in the observed age group, the training protocol of the junior elite orienteering athletes was generally adequate. In comparison to elite track, marathon, and orienteering athletes, the junior elite athletes performed less high-intensity interval training (HIIT). However, more frequent HIIT seems to be a protective factor against injuries.

## 1. Introduction

Monitoring training is indispensable for competitive athletes who aspire to excel at elite levels. Based on a systematic collection of training data, planned and performed training content and intensity can be compared [1, 2]. Further, monitoring training supports holistic training regulations and is, therefore, important to enable optimal individual training adaptations [3]; hence, prevention or early detection of musculoskeletal injuries might be possible [4]. Since musculoskeletal injuries are a major reason for premature career termination in elite athletes [5–7], every effort to use training monitoring data for injury prevention is paramount. Musculoskeletal injuries are generally defined as injuries or disorders of the muscle, tendon, bone, joint, ligament, or nerves [8, 9]. Injuries can be defined further by the location, type, grade of injury, or training days lost due to an injury [10–12]. Injuries not only pose a problem to the physiological system but can also be a psychological burden, limiting the preparation time for the competition season [12]. Characteristics of musculoskeletal injuries in running-related sports have been widely investigated and varying injury incidence rates (IIRs) per 1000 hours of training have been reported. In van Mechelen's review [13], 2.5–12.1 injuries per 1000 hours of running were reported for recreational and competing runners. Other authors have reported a similar range of IIRs between 1.7 and 10.0 injuries per 1000 hours of running exposure [14, 15]. In a recent review, an IIR range of 2.5–38.0 was reported [16]. Comparisons between studies that assess IIR must be made with caution due to the different definitions of running-related injuries, the subjects included, and the methods used for injury assessment [9]. Competitive and elite athletes tend to have fewer IIRs per 1000 hours of exposure than recreational or inexperienced athletes [13, 15]. In a study with young competitive orienteering athletes, 3.0 injuries per 1000 hours of training were reported [12].

Orienteering is a running-related endurance sport comprised of physical and cognitive components. Orienteering athletes compete on a timed run through unknown cross-country terrain, checking in at predetermined control sites while navigating with only a map and compass [17, 18]. The most common events are sprint and middle and long distance races, with completion times of 15–20, 40–50, and 70–90 minutes, respectively. Different competition modes exist; however, most often the athletes start individually within short time intervals [11, 19]. Switzerland has a history of successful orienteering athletes. The final ranking of the 2014 International Orienteering Federation World Cup [19] displayed Switzerland as the best nation, with 5 women classified in the top 12 and 7 men in the top 15. Due to the impressive international success of Swiss orienteering athletes, an investigation of their injury and training data is of great interest.

Recently, training data of Norwegian elite orienteering athletes were published. Tønnessen et al. [20] reported the annual training periodization of eight Norwegian world champions in orienteering. During their most successful seasons, leading to a win at the World Orienteering Championship, the athletes recorded 9-10 training sessions and 10.6–14.9 hours of training duration per week. A significant reduction in training duration from general preparation to specific preparation and competition phase was observed. This was primarily due to a reduction of training sessions of low-to-moderate intensities, whereas the high-intensity interval training (HIIT) duration was increased [20]. An older study with female elite orienteers reported 3–7 training sessions per week, with 63% of squad members training between 5.0 and 8.9 hours per week [11]. More research is available for elite track and marathon runners, reported to train 10–13 training sessions per week and running an average of 126–186 km per week [21, 22].

Little is known about the relation between such training data and injury occurrence in the running-related sport of orienteering. Therefore, the aim of this study was to investigate the relationship between the data from the athletes' training diaries recorded at the ages of 18 and 19 years and injury occurrence in Swiss junior elite orienteering athletes.

## 2. Materials and Methods

### 2.1. Study Design and Selection of Participants

A retrospective study design was chosen in cooperation with the Swiss Orienteering Federation, which has been monitoring the injuries, illnesses, and performance changes of their elite athletes for decades. In this study, currently active elite athletes and athletes active in the Swiss Orienteering Federation in the last 20 years were asked to provide their individual training diaries. Inclusion criteria were currently 18+ years of age and selection for the junior or elite national team at one point during their athletic career. The volunteer subjects gave written informed consent after receiving written and oral documentation about the study's purpose. The data collection was conducted in accordance with the Declaration of Helsinki [23] and approved by the local ethics committee. The age group of 18- and 19-year-old athletes, for which the most training data was provided, was chosen for the data analysis.

### 2.2. Training Diary and Medical History

The athletes provided their training diaries in digital or paper-pencil versions as far retrograde as available. Every training session recorded in a paper-pencil training diary was digitized. Afterwards, the variables were summarized for each training week. With the weekly data, the individual annual mean was calculated per age group. The variables were total duration, duration, total frequency, and frequency for endurance running, HIIT, orienteering, strength training, complementary endurance training, and regeneration, with warm-up and cool-down and stretching subcategories included in the duration variable, training load, monotony in total duration, monotony in training load, and proportion of HIIT within endurance running (Table 1). Training load was computed according to Foster et al. [24] and Edwards [25]. A factor was assigned to each training intensity category (regenerative = 1; extensive = 2; moderate = 3; intensive = 4; supramaximal = 5). To calculate the training load, training duration in each intensity category was multiplied with the corresponding factor and summarized. Monotony in weekly training duration was computed as “mean duration per week” divided by the “standard deviation duration per week.” Similarly, monotony in training load was computed as “mean training load per week” divided by the “standard deviation training load per week” [26]. The proportion of the HIIT within all endurance running training sessions was computed as “duration of HIIT” divided by the “sum of the duration of HIIT and endurance running.”

Injuries and illnesses were assessed from the athletes' personal medical records. The medical doctors of the Swiss Orienteering Federation maintained these records. All entries concerning disorders of the musculoskeletal system, concussions, or illnesses to other body systems were included. Illnesses were classified using the International Classification of Primary Care, Second Edition (ICPC-2) [27]. For injury classification, the Orchard Sports Injury Classification System version 10 (OSICS-10) was used [28]. The first letter of the code determined the location of the injury and the second letter defined the type of injury. Further, injuries were discriminated into acute and overuse injuries. Acute injuries were caused by a sudden single traumatic event, whereas overuse injuries originated from repetitive overload of the same musculoskeletal structures without a clearly identifiable event. Each injury's severity was classified as trivial (no consequences), minimal (1–3 training days lost, TDL), mild (4–7 TDL), moderate (8–28 TDL), severe (>28 TDL), or ultimate (end of athletic career due to injury or permanent physical damage) [29]. The injury incidence rates (injuries per 1000 training hours) were calculated as “number of total injuries” divided by “training hours” multiplied by 1000. The injury incidence proportion (%) was calculated as “number of athletes with ≥1 injury” divided by “total number of athletes” multiplied by 100. For the analysis of the effect of training patterns on injuries, an injury severity index was computed for each athlete as “number of injuries per year” multiplied by “maximal severity.”

### 2.3. Competition Results

The individual and relay achievements during World Orienteering Championships (WOC), European Orienteering Championships (EOC), Junior World Orienteering Championships (JWOC), European Youth Orienteering Championships (EYOC), and the World Cup (WC) were collected partly from the results archive of the International Orienteering Federation [19] and were partly provided from the database of the Swiss Orienteering Federation. Athletic success was defined as at least one medal at a WOC or EOC or a top 3 result in the WC some time during the athlete's career. Hence, the participating athletes' individual and relay competition results from their first competition to the end of 2014 were included for the analysis.

### 2.4. Statistical Analysis

IBM SPSS Statistics 22 for Windows (IBM Corporation, Armonk, NY, USA) was used for the statistical analysis. The level of significance was set at *α* = 0.05.

The data of all athletes providing training data at the age of 18-19 years were used for analysis. The data were tested for normal distribution. Group results are presented as mean ± standard deviation. A multiple linear regression with stepwise backward elimination was applied to identify predictor variables for injuries. The injury severity index was set as a dependent variable. The independent variables were gender, previous injuries, mean total duration, mean total frequency, mean duration, and mean frequency of each training category, for example, endurance running, HIIT, orienteering, strength training, complementary endurance training, and regeneration, respectively, and mean warm-up and cool-down duration, mean training load, mean monotony in total duration, mean monotony in training load, and proportion of HIIT within all endurance running. The included independent variables were previously tested for colinearity using Pearson's correlation coefficient. Furthermore, differences between gender, internationally successful and nationally successful athletes, and athletes with and without previous injuries were tested using a Mann-Whitney test.

## 3. Results

Data from the athletes aged 18 and 19 years were available for 31 (15 women, 16 men, current mean age 22.45 ± 4.01 years) out of the 43 volunteering athletes. The participating athletes achieved a combined 124 first places, 42 second places, and 30 third places at international orienteering championships (WOC and EOC) and in WC competitions during their active careers.

The training characteristics of the Swiss junior elite orienteering athletes are reported in Table 1.

The reported injuries in the athletes' medical records are presented in Table 2. Of the 61 injuries, 44 (72.13%) were due to overuse and 17 (27.87%) injuries were a consequence of an acute trauma. Injury incidence rate per 1000 hours of training was 2.18 ± 2.13 for all injuries. For overuse injuries, it was 1.51 ± 1.78, and for acute injuries, it was 0.54 ± 1.12 injuries per 1000 hours of training. The injury proportions were 77.42%, 70.97%, and 35.48% for all, overuse, and acute injuries, respectively. The lower extremity was affected in 93.44% of the reported cases, whereas the upper extremity was injured in 6.56% of cases. No injuries to the head, neck, trunk, chest, pelvis, buttocks, or thigh were registered (Table 2). About 50% of the athletes had experienced an injury in the previous year and in 24.59% of cases the medical problem was a recurring injury.

To investigate the influence of training patterns on the injury severity index, a multiple linear regression analysis was performed. Of all the independent variables included in the multiple linear regression analysis, seven predictor variables remained in the final regression model (Table 3). Together, these variables explained 59.66% of the variance in the injury severity index between the individual athletes (*R* ^2^ = 0.650, *R* ^2^ adjusted = 0.597, *F*(7.46) = 12.200, *p* < 0.001). Male gender, low duration and high frequency of endurance running, low frequency of HIIT, high duration of complementary endurance training and regeneration, and high monotony in total duration are significant predictor variables for an increased injury severity index.

The Mann-Whitney test reported significant differences for gender, athletic success, and previous injury history. The male athletes reported significantly fewer training sessions per week (6.89 ± 1.56 versus 7.87 ± 2.29 for male and female athletes, resp.; *p* = 0.013). Looking more closely at the content, the men performed strength training significantly less frequently than the female athletes (1.03 ± 0.50 versus 1.40 ± 0.63 training sessions/week, resp.; *p* = 0.009). Further, male athletes had a significantly higher mean monotony in total duration compared to female athletes (3.44 ± 1.50 versus 2.83 ± 0.40, resp.; *p* = 0.034). Regarding athletic success, the training patterns revealed that the less successful athletes generally trained longer (474.26 ± 95.61 versus 417.78 ± 94.78 min/week, resp.; *p* = 0.046) and more frequently (7.80 ± 2.13 versus 6.52 ± 1.41 training sessions/week, resp.; *p* = 0.035) and, specifically, did more frequent HIIT (0.47 ± 0.23 versus 0.33 ± 0.19 training sessions/week, resp.; *p* = 0.026) compared to the internationally successful athletes. Further, the more successful athletes had achieved significantly more top 3 results at the junior elite level at JWOC and EYOC competitions than less successful athletes (*p* = 0.005). The previous injury variable was just short of being significant as well (*p* = 0.055). About one-third of the internationally successful athletes experienced a previous injury, whereas, of the less successful athletes, almost two-thirds had previous injuries; however, this variable was not significantly different for the two tested groups. Regarding the effect of previous injury history, athletes with at least one injury in the previous season injured themselves significantly more during the investigated training period than the previously uninjured athletes (1.32 ± 1.14 versus 0.71 ± 0.85 injuries, resp.; *p* = 0.020). The orienteering athletes with a previous injury reported longer (17.62 ± 19.77 versus 7.60 ± 13.16 min/week, resp.; *p* = 0.024) and more frequent regeneration activities (0.51 ± 0.76 versus 0.30 ± 0.71 training sessions/week, resp.; *p* = 0.046) and performed more frequent orienteering training sessions (2.40 ± 0.85 versus 1.91 ± 0.61 training sessions/week, resp.; *p* = 0.024) than the previously uninjured athletes.

## 4. Discussion

### 4.1. Training Patterns

The orienteering athletes reported 7.59 ± 1.64 hours per week total training duration distributed into 7.37 ± 2.00 training sessions. This amount of weekly training is comparable to an earlier study with female orienteering athletes on the Great Britain national team, where 63% of the athletes ran 5–9 hours per week [11]. However, the current training duration is shorter than the reported 10–15 hours per week in a study with Norwegian elite orienteers [20]. The reason for this discrepancy is probably related to the age of the athletes. The Norwegian athletes were at the height of their careers and were six or seven years older than those in the age group that was analyzed in the current study. A direct comparison with track and marathon runners is not possible due to the different assessments of training volume (time versus distance). However, the training frequency can be compared. Different authors have reported 9–12 or 11–14 training sessions per week for male and female elite track and marathon runners [21, 22]. The athletes in the current study reported a slightly lower number of training sessions per week, but this is in accordance with the training frequency of 9-10 sessions per week for Norwegian elite orienteering athletes [20].

The Swiss junior elite orienteering athletes performed 11% of their running training as HIIT, and the rest was spent on low-to-moderate intensity endurance runs. Recently, studies with male and female elite track and marathon runners [21, 30, 31] and elite orienteering athletes [20] assumed the optimal proportion of HIIT within all endurance running sessions to be 20%. These highly trained athletes performed 80% of their training at low intensities (62–82% of the maximal heart rate) and the remaining 20% at high intensities (82–92% of the maximal heart rate), with favorable effects on their running performance [21]. In the current study, the elite orienteering athletes in the age group of 18-19 years reported a lower percentage of HIIT. Based on the previously reported results, it might be assumed that the orienteering athletes in the current study should have performed HIIT more frequently, at the expense of other training sessions. This statement is further supported from the aspect of injury prevention. The multiple linear regression analysis showed that athletes who do HIIT more frequently suffer fewer or less severe injuries. Similarly, Hespanhol Jr. et al. [14] reported HIIT to be a protective factor against running-related injuries.

### 4.2. Injury Location and Type

A total of 61 injuries were reported in the examined age group. The most common injury location was the knee (33%), which is in accordance with the injury patterns reported in the literature for runners and orienteering athletes [12–14, 32–34]. Thereafter, injuries occurred most often at the lower leg (21%), the ankle, and the feet (both 18%). The lower extremity was affected in 93% of the cases, demonstrating the strenuous demands that running-based sports put on the lower extremities [12, 13]. Overuse injuries were diagnosed in 72% of the cases, and only 28% were due to an acute trauma. These results are consistent with previously reported injury data for runners and orienteering athletes [10, 12, 13]. Injury type classification revealed 46 (75%) of all injuries classified as inflammation and/or pain. Further, five (8%) sprains, five (8%) dislocations, one (2%) fracture, and one (2%) laceration were diagnosed. The remaining three injuries (5%) were described as bruising. Of the 61 injuries, 49 injuries (80%) were classified as trivial. The remaining injuries were of minimal (10%), mild (3%), and moderate (7%) severity. Johansson [12] investigated injuries in young athletes in a Swedish orienteering college over a year. Injury severity was reported as 20% mild, 60% moderate, and 20% severe. This injury distribution is of greater severity than the injuries observed in the current study, which were mainly trivial and minimal. No severe or ultimate injuries were assessed in the Swiss elite orienteering athletes in the age group of 18-19 years. The reported IIR of 2.18 ± 2.13 injuries per 1000 hours of training was rather low compared to previous publications [13–16]. However, elite athletes generally reported lower IIRs compared to recreational or inexperienced runners and an orienteering-specific study reported a similar IIR of 3.0 injuries per 1000 hours of training [12, 13]. The lower IIR of orienteering athletes might be a consequence of the different training distribution in the running-related orienteering sport compared to track and marathon running. In orienteering, approximately 50% of the training duration is spent on running activities, whereas marathon runners perform more running sessions and approximately 80–85% of all training sessions are spent on running activities [21, 22]. Therefore, the training variety of orienteering athletes is greater, which may be the reason for the lower IIR.

### 4.3. Training Patterns and Injury Severity Index

When computing a multiple linear regression with injury severity index as the dependent variable, gender, duration and frequency of endurance running, frequency of HIIT, duration of complementary endurance, duration of regeneration, and monotony in total duration explained 59.66% of the variance in individual injury severity indices. These variables are discussed in the following section, starting with gender.

The present data showed that male orienteering athletes suffered from relatively more or more severe injuries than their female peers in the present population. A previous study reported a higher Achilles tendon injury risk for recreational male runners compared to female runners [35]. However, other studies reported higher injury rates in female runners or conflicting results for gender differences [9, 34, 36, 37]. A short duration and a higher frequency of endurance running led to a greater injury severity index. Several previous studies reported that runners with a high weekly running mileage are at greater risk of sustaining an injury [9, 10, 13, 15, 16]. However, the results for running frequency are not as explicit. Training frequency seems to follow a u-shape in injury risk: too few and too many training sessions per week were associated with an increased injury risk [13, 16, 34, 36, 37]. The present results suggest that endurance running should be performed less frequently but as longer runs. The injury severity index correlated negatively with HIIT frequency. The mean overall duration of a HIIT session was 28.88 ± 16.56 minutes, divided into multiple intervals (15 seconds up to several minutes) and recovery duration (15–180 seconds). The shorter the interval duration, the greater the number of repetitions (generally 2–10 repetitions). If the athletes performed more than one round of HIIT, breaks of 2–4 minutes were common between rounds. The athletes in this study executed an average of 11% HIIT during their running activities. Regarding optimal performance improvements, previous studies with elite runners reported that 20% of the training volume should be performed as HIIT to achieve desirable performance changes [21]. The results of the present study suggest that this proportion is optimal for injury prevention as well. The mean proportion was 11% HIIT, and athletes who achieved higher HIIT percentages reported fewer, less severe, or no injuries. Surprisingly, orienteering athletes with greater durations of complementary endurance training and more time spent with regeneration activities had a higher injury severity index. An explanation could be that athletes who suffered from a running-related injury tended to perform more complementary endurance and regeneration training compared to noninjured peers. Therefore, the directionality of the causality between the injury severity index and complementary endurance and regeneration training, respectively, remains unclear. However, when the subgroup of athletes with at least one previous injury was compared to the one with athletes without previous injuries, the previously injured athletes reported significantly longer and more frequent regeneration procedures. Therefore, the assumption that previously injured athletes do have an increased need for regeneration or they are more sensitized to the gain of regeneration activities can be supported. Finally, a high monotony in total duration index was positively associated with a higher injury severity index. Anderson et al. [4] and Foster [26] previously observed similar results with monotony in training load in collegiate and elite athletes. Therefore, the current results support the notion that the daily training load should be distinctly different in order to minimize training monotony and the associated increased injury risk [26]. In the present study, male athletes had a higher monotony index for their weekly training. This might partly explain the results that male orienteering athletes were significantly more often or more severely injured than female athletes. Previous injuries were repeatedly reported as a risk factor for injury occurrence in runners [9, 10, 13, 14, 36]. However, previous injuries were not associated with a greater injury severity index in the current population of orienteering athletes.

The male athletes performed significantly fewer weekly training sessions in general and fewer strength training sessions specifically than the female athletes. Further, the male athletes had a greater monotony in total duration index. Since male athletes suffered more injuries, it seems that a certain amount of strength training, 2-3 training sessions per week, is beneficial for injury prevention.

When comparing internationally successful and nationally successful athletes, significant differences were observed in total duration, total frequency, and frequency of HIIT. The less successful athletes performed significantly longer and more training sessions as well as more HIIT sessions per week. Looking at previous injuries, the athletes who achieved international success during their careers tended to have fewer injuries in the 18-19 years' age group; however, this difference was not statistically significant. It seems that the less successful athletes had to train more to achieve similar results compared to the more successful athletes, since the more successful athletes achieved significantly more top 3 rankings already at the junior elite level championships (JWOC and EYOC) compared to the less successful athletes when aged 18-19 years.

### 4.4. Strength and Limitations

Scientific studies with elite athletes normally consist of small sample sizes due to limited time and participants and compliance with training protocols. Therefore, a benefit of this study is the inclusion of data from 31 internationally and nationally successful athletes. The chosen retrospective design makes it possible to minimize the recall bias concerning training characteristics by using daily recorded data instead of questionnaires answered by the athletes many years later.

The individual training periods in the age group of 18-19 years span almost two decades; however, Tønnessen et al. [20] reported in a recent study that the training distribution in orienteering did not change profoundly between 1979 and 2012. Therefore, it seems feasible to compare the athletes in the age group of 18-19 years. Further, the current mean age of the participants (22.45 ± 4.01 years) indicates that most athletes only recently belonged to the observed age group. As a limitation of this study, the way the athletes handled their training diaries varied. Most athletes reported every single training session and distinguished between warm-up, strengthening unit, technique training, and main training session, while some mentioned the main content and the total duration of a training session only. Further, it is likely that not all injuries, especially trivial-to-mild injuries, were entered into the medical records. Some athletes might not have contacted their medical doctor about trivial injuries because the injury did not result in loss of training days or require special treatment. However, the coverage of moderate-to-ultimate injuries is complete due to the documented communication between the medical doctors of the Swiss Orienteering Foundation and the attending specialized doctors. Unfortunately, only few orienteering athletes recorded the distance or altitude covered during training sessions. Similarly, most athletes did not record the rate of perceived exertion or subjective load after a training session. Hence, no such data was included in the analysis and the comparability to similar studies is limited.

## 5. Conclusion

The reported training loads of Swiss junior elite orienteering athletes aged 18 and 19 years are comparable to the training loads of elite runners and orienteering athletes in other studies. However, the training patterns cannot be compared between these studies because detailed results are missing in previous studies. Thus, it is difficult to make direct comparisons. The orienteering athletes seem to apply a reasonable training protocol, as there were a few, and notably no severe or ultimate injuries are observed in this group of athletes. The injury incidence rate is at the lower end of previously reported rates. Further, the injury patterns assessed in junior elite orienteers match those of runners in general and orienteers in particular.

This study showed the relation between HIIT and injury severity. To prevent running-related injuries in Swiss elite orienteers, more HIIT sessions should be performed in comparison to all running training sessions. Additionally, male orienteering athletes need to reduce the monotony in their training duration index and should include more strength exercises in their training program. To achieve that, athletes and coaches are advised to monitor daily training using measurable variables such as duration, frequency, intensity calculated by physiological values or time, and the distance covered. To improve the monitoring of orienteering training and to minimize the training monotony, orienteering athletes should also more frequently report subjective values such as the rate of perceived exertion in order to calculate session training load. Finally, these results should be confirmed through prospective intervention studies on training and injury patterns in elite athletes.

## Figures and Tables

**Table 1:** Training characteristics of Swiss junior elite orienteering athletes aged 18 and 19 years.

Variable	Subcategory	Unit	Training data
Duration	Total duration	min/week	455.75 ± 98.22
	Endurance running	min/week	108.00 ± 32.88
	HIIT	min/week	12.42 ± 7.12
	Orienteering	min/week	99.31 ± 36.87
	Strength training	min/week	49.90 ± 29.59
	Complementary endurance	min/week	90.03 ± 63.97
	Regeneration	min/week	12.95 ± 17.61
	Warm-up/cool-down	min/week	64.50 ± 20.42
	Stretching	min/week	31.59 ± 21.47

Frequency	Total frequency	TS/week	7.38 ± 2.00
	Endurance running	TS/week	2.23 ± 0.76
	HIIT	TS/week	0.43 ± 0.23
	Orienteering	TS/week	2.17 ± 0.78
	Strength training	TS/week	1.22 ± 0.60
	Complementary endurance	TS/week	1.32 ± 1.02
	Regeneration	TS/week	0.42 ± 0.74

Training load		Index	754.68 ± 192.20

Monotony in total duration		Index	3.13 ± 1.13 (range 1.54–10.18)

Monotony in training load		Index	2.80 ± 0.75 (range 1.32–4.82)

Proportion of HIIT within endurance running		%	10.52

*Note*. HIIT = high-intensity interval training. TS = training session. Complementary endurance includes cross-country skiing, cycling, and swimming. Regeneration includes treatments such as massages, physiotherapy, and sauna, and the duration of these treatments was not included in total duration per week.

**Table 2:** Injury occurrence in Swiss junior elite orienteering athletes.

		Number	%
Injury location	Upper extremity	4	6.56
	Hip/groin	2	3.28
	Knee	20	32.79
	Lower leg	13	21.31
	Ankle	11	18.03
	Foot	11	18.03

Injury type	Inflammation and pain	46	75.41
	Sprain	5	8.20
	Dislocation	5	8.20
	Fracture	1	1.64
	Laceration	1	1.64
	Bruising	3	4.92

Injury severity	Trivial	49	80.32
	Minimal	6	9.84
	Mild	2	3.28
	Moderate	4	6.56
	Severe	0	—
	Ultimate	0	—

*Note. *Upper extremity includes shoulder, upper arm, elbow, forearm, wrist, and hand injuries. Trunk and chest include trunk, chest, abdominal, and spine injuries.

**Table 3:** Multiple linear regression with stepwise backward elimination to predict training variables influencing injury severity index.

Predictor variable	*B*	SE *B*	*β*	*T*	*p*
Gender [male, female]	−1.107	0.511	−0.215	−2.167	0.035
Duration of endurance running [min/week]	−0.030	0.014	−0.381	−2.183	0.034
Frequency of endurance running [TS/week]	2.708	0.679	0.789	3.985	<0.001
Frequency of HIIT [TS/week]	−5.555	1.425	−0.464	−3.898	<0.001
Duration of complementary endurance [min/week]	0.016	0.005	0.373	3.290	0.002
Duration of regeneration [min/week]	0.048	0.020	0.297	2.376	0.022
Monotony in total duration [index]	1.305	0.436	0.297	2.993	0.004
Constant	−2.910	2.165		−1.344	0.185

*Note.* TS = training sessions; HIIT = high-intensity interval training.
